# Facile synthesis of urchin-like RuCu and hollow RuCuMo nanoparticles and preliminary insight to their formation process by cyclic voltammetry†

**DOI:** 10.1039/c8ra01261j

**Published:** 2018-04-17

**Authors:** Yanna Song, Jingcheng Sun, Yanru Zhang, Bingxin Wang, Qiang Li, Yongming Fan

**Affiliations:** MOE Engineering Research Center of Forestry Biomass Materials and Bioenergy, Beijing Forestry University Beijing 100083 China; Key Laboratory of Lignocellulosic Chemistry, Beijing Forestry University Beijing 100083 China fanym@bjfu.edu.cn +86-185-15301003; College of Science, Beijing Forestry University Beijing 100083 China liqiang@bjfu.edu.cn +86-137-18679671

## Abstract

Urchin-like RuCu nanoparticles and hollow RuCuMo nanoparticles were prepared by a one-pot chemical reduction method. The nanoparticles were characterized by EDX, HRTEM, XPS and ICP-AES. By combining cyclic voltammetry and TEM, the formation process of nanoparticles was obtained. The urchin-like RuCu nanoparticles are proved to be formed *via* underpotential deposition mechanism and the formation of ternary nanoparticles RuCuMo was due to the replacement of Cu with Ru and the interception of Mo gradually. It was found that the formation of different morphology is depended on the precursors in the reaction system and their reduction sequences. Compared to previously reported multi-step synthetic routes, the developed method here is much simpler.

## Abstract 2

The urchin-like RuCu and hollow RuCuMo nanocrystals were synthesized by one-pot chemical reduction method. The formation process of these nanocrystals was traced by cyclic voltammetry.

## Graphical abstract

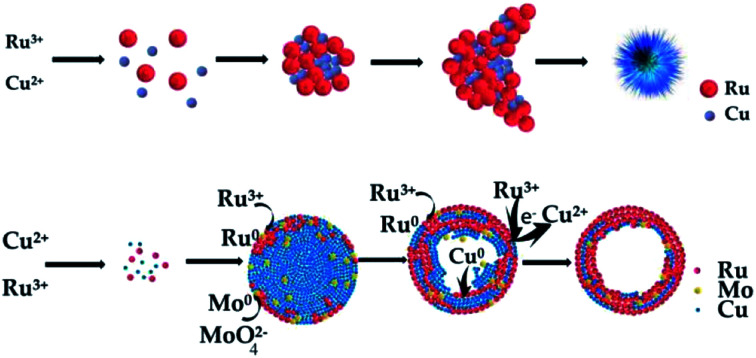

## 1. Introduction

The synthesis of multi-component metallic nanoparticles (NPs) has been a hot topic in the past decades because of their various important applications, such as optics, bioanalysis and catalysis.^1–5^ Due to electronic and synergistic effects, multi-component metallic NPs often show unique catalytic activities and superior selectivity and stability to monometallic counterparts. It has been reported to result from the introduction of transition metals (*i.e.*, Cu, Co, Ni, *etc.*) into noble-metal NPs, which enhances the catalytic activity and decreases the consumption of noble metals. Shevchenko *et al.*^6^ firstly reported the colloidal synthesis of monodisperse CoPt_3_ NPs with different sizes and high stability. Many efforts have been made therewith for the synthesis of NPs by solution reduction,^7–9^ electrochemical synthesis,^10^ and hydrothermal synthesis.^11^ However, the Ru-based NPs has been rarely reported. As an important platinum-group metal, Ru is comparatively cheaper and has been widely used in many important reactions such as CO oxidation,^12^ selective hydrogenation,^13^ and dehydrogenation of ammonia borane.^14^ Despite successful synthesis of Ru NPs with well-defined size and morphology,^15–21^ the synthesis of Ru-based NPs received limited success.^22,23^ A possible reason for this is that Ru is too active to be stable in oxidative environment. The introduction of a second or third party onto Ru could be a solution for this challenging problem. However, the synthesis of ternary Ru-based NPs has not been reported so far.

Understanding of the formation process of NPs is necessary for the design of catalyst with targeted properties.^22^ Zhu *et al.* have reported the synthesis of PtNi nanocrystals by tracing the Ni contents in particles with TEM and EDX at different reaction stages.^24^ Polte and his co-workers have presented the formation mechanism of gold NPs with the help of *in situ* small-angle X-ray scattering and X-ray absorption near-edge spectroscopy.^25^ However, it still remains challenging to figure out the time-dependent characterization of the formation process of those NPs.

Herein, a facile and effective approach was introduced to synthesize urchin-like RuCu NPs and hollow RuCuMo NPs. The products were characterized by TEM, HRTEM, XPS and ICP-AES and the formation process was monitored by measuring the cyclic voltammetric behaviors.

## 2. Materials and methods

### 2.1 Materials

Ruthenium(iii) acetylacetonate (Ru(acac)_3_, 99%) were purchased from Beijing HWRK Chem Co., LTD. Copper(ii) acetylacetonate (Cu(acac)_2_, 95%) were purchased from J. K Scientific. Poly(vinyl pyrrolidone) (PVP, MW 22000) were purchased from Xilong chemical. Sodium molybdate (Na_2_MoO_4_, 95%), and benzyl alcohol (C_6_H_5_CH_2_OH, ≥99.9%) were all purchased from Sigma-Aldrich. These chemicals were pure without further processing.

### 2.2 Synthesis of urchin-like RuCu NPs

A series of control experiments under different reaction conditions were conducted. And the optimum conditions are as follows: Ru(acac)_3_ (0.04 mmol), Cu(acac)_2_ (0.04 mmol) and PVP (71 mg) dispersed in C_6_H_5_CH_2_OH (7.50 mL) were added into a vial (volume: 30 mL). After the vial had been capped, the mixture was sonicated for about 5 minutes. The resulting homogeneous mixture was then heated at 160 °C for 8 h in an oil bath and then cooled down to room temperature after reaction. The resulting colloidal products were collected and washed five times with acetone by centrifugation.

### 2.3 Synthesis of hollow RuCuMo NPs

A series of control experiments under different reaction conditions were conducted. And the optimum conditions are as follows: Ru(acac)_3_ (0.04 mmol), Cu(acac)_2_ (0.04 mmol), Na_2_MoO_4_ (0.04 mmol) and PVP (71 mg) dispersed in C_6_H_5_CH_2_OH (7.50 mL) were added into a vial (volume: 30 mL). After the vial had been capped, the mixture was sonicated for about 5 minutes. The resulting homogeneous mixture was then heated at 160 °C for 8 h in an oil bath and then cooled down to room temperature after reaction. The resulting colloidal products were collected and washed five times with ethanol by centrifugation.

### 2.4 Characterizations

The transmission electron microscope (TEM) images were taken using a Hitachi-7700 microscope operated at 100 kV. The energy dispersive X-ray (EDX) analysis and high-angle annular dark-field scanning TEM (HAADF-STEM) analysis were performed using high resolution transmission electron microscopy (HRTEM, JEM 2100F) at 200 kV. The samples for TEM observation were prepared by dispersing the metal nanoparticles in ethanol at room temperature and then transferred on a carbon-coated nickel grid. The size distribution of 150 NPs was measured with Nano Measurer software. X-ray photoelectron spectroscopy (XPS) experiments were performed on PHI-5300 Quantera microprobe. The element composition of NPs was determined by the inductively coupled plasma atomic emission spectroscopy (ICP-AES, IRIS Intrepid II).

### 2.5 Cyclic voltammetry analysis

The cyclic voltammetry analysis were performed using a CHI 660E electrochemical analyzer (CH Instrument, Chenhua Co., Shanghai, China) in a conventional three-electrode system including an Ag/AgCl (saturation KCl) reference electrode, a glassy carbon working electrode, and a platinum wire counter electrode. The glassy carbon electrode was polished with slurry of 0.5 μm and 0.05 μm alumina to mirror, ultrasonically washed in ethanol and deionized water for 20 min and dried at room temperature. The catalyst nano powder was suspended in 1 mL ethanol and 50 μL Nafion solution (5%) in ultrasonic for 20 min to make the homogeneous catalyst ink. The 10 μL catalyst ink was dropped onto the clean surface of the glassy carbon electrode. It is dried at room temperature to form a working electrode. The CV measurements are performed in N_2_-saturated 0.5 M H_2_SO_4_ solution at a scanning rate of 10 mV s^−1^. The CV curves were measured by running 500 times to ensure reproducibility.

## 3. Results and discussion

**Fig. 1:**
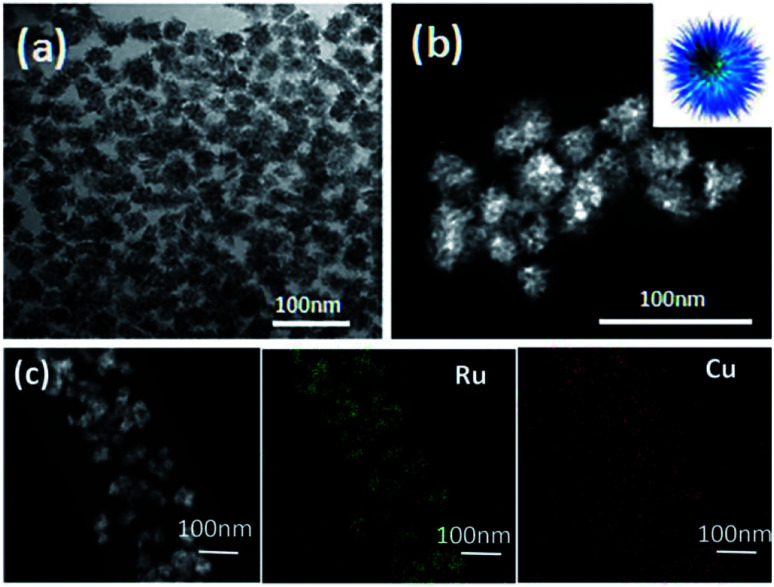
(a) TEM and (b) HAADF-STEM images of urchin-like RuCu (the inset in (b) is a schematic illustration of urchin-like RuCu); (c) EDX elemental mapping images.

TEM and HAADF-STEM images were taken to probe the morphology of the as-synthesized RuCu sample. As shown in Fig. 1, the most of the particles are in urchin-like shape. The diameter of the particles ranges from 21 nm to 33 nm (Fig. 1a and S1, ESI†). The EDX elemental mapping (Fig. 1c) and line-scanning images (Fig. S2a, ESI†) showed the structure of RuCu NPs. It was revealed that Ru and Cu element are uniformly distributed on the entire NPs, implying the formation of RuCu alloy. Both the EDX (Fig. S2b, ESI†) and ICP-AES analysis displayed the similar Ru : Cu atomic ratio of about 0.69 : 0.31. This atomic ratio was higher than the Ru/Cu stoichiometric ratio in the reaction system (Ru(acac)_3_/Cu(acac)_2_ (1 : 1)), which could be caused by the dissolution of un-alloyed Cu during washing step. The signal of Ni in Fig. S2b (ESI†) was originated from the nickel grid used in the measurement. The HRTEM images of RuCu NPs shows some similar urchin-like picture. The SAED image shows well crystallinity and the polycrystal structure (Fig. S3, ESI†).

The XRD pattern of the RuCu NPs was shown in Fig. S4 (ESI†). By comparing the XRD pattern of pure Ru standard card (JCPDS card no 06-0663), we found that the diffraction peaks of the prepared nanoparticles were all right-shifted, which were supposed to be caused by the addition of Cu in Ru crystal lattice, which changed the inter-planar spacing. The characteristic peaks of RuCu were between the two single metal peaks, indicating that the obtained material was RuCu alloy. The diffraction peaks of RuO_2_ (110) (2*θ* = 28.0°) (JCPDS card no 43-1027) appeared in Fig. S4 (ESI†) suggest the presence of Ru oxidation state (RuO_2_).

**Fig. 2:**
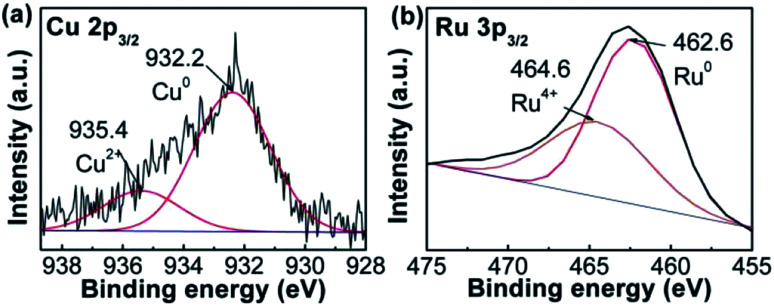
(a) Cu 2p_3/2_ and (b) Ru 3p_3/2_ XPS spectra of RuCu nanoparticles.

The compositions of RuCu sample were studied using XPS. The spectrum was separated into two peaks (Fig. 2a). The peak at ∼932.2 eV is attributed to Cu^0^, while that at ∼935.4 eV corresponds to Cu^2+^ in RuCu NPs. The content of 21.7% Cu^2+^ in RuCu NPs was based on the peak area. The same protocol was applied to Ru 3p_3/2_ spectrum (Fig. 2b). The peak at ∼462.6 eV was attributed to Ru^0^ and that at ∼464.6 eV was assigned to Ru^4+^ (RuO_2_),^26^ which is assistent with XRD result. 26.9% of Ru^4+^ remained in the RuCu NPs.

In the control experiment using only Ru precursor in the synthesis, 7 nm Ru particles with uncontrollable shape are obtained (Fig. S5, ESI†), while using only Cu precursor, even no particles were found. However, in the case of coexisted precursors of Ru and Cu in the synthesis, the urchin-like RuCu NPs were achieved. This suggested that coexistence of Ru and Cu was essential for the synthesis of urchin-like particle.

**Fig. 3:**
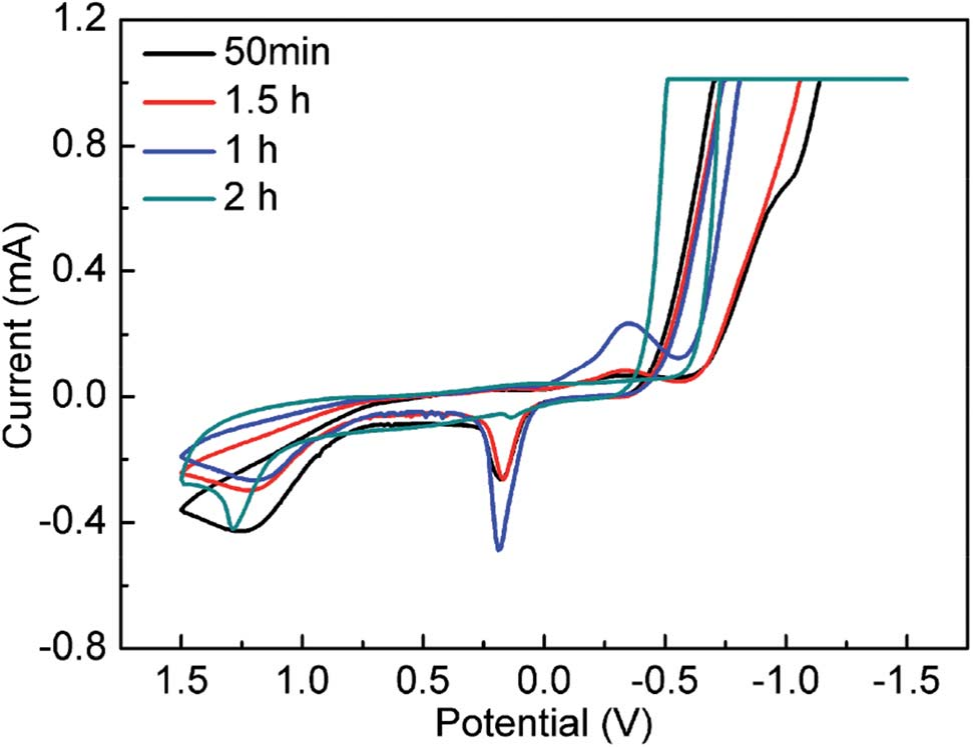
The CV curves at different RuCu nanoparticles sampling time.

To study the formation process of NPs, cyclic voltammetry (CV) measurement was employed. CV curves corresponding to 50 min, 1 h, 1.5 h, and 2 h respectively were showed in Fig. 3. The typical CV curves of ruthenium, copper, molybdenum NPs, RuCu and CuMo bimetallic NPs were obtained and displayed in Fig. S6 (ESI†). The CV curve at 50 min is consistent with that of Ru NPs (Fig. S6b, ESI†) and the CV curve at 2 h is agreement with that of RuCu NPs (Fig. S6d, ESI†), suggesting that Ru^3+^ is reduced prior to Cu^2+^ during the reaction. Theoretically, the standard redox potential of Ru^3+^/Ru^0^ and Cu^2+^/Cu^0^ is 0.68 V and 0.34 V respectively, so the reduction of Ru species should be easier than Cu species.^27^

**Fig. 4:**
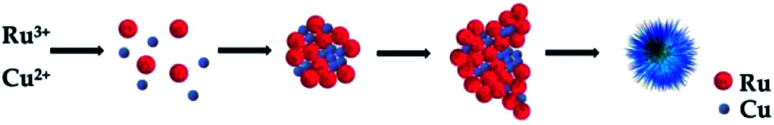
Schematic illustration of the formation process of RuCu bimetallic nanoparticles.

Based on the results above, we postulated the formation process of RuCu NPs (Fig. 4). At the initial stage, Ru^3+^ and Cu^2+^ are reduced by benzyl alcohol but Ru atoms tend to be reduced in a faster rate than Cu and nucleate, grow first. At the second stage, by way of underpotential deposition effect,^28^ the produced Cu atoms are deposit on the formed Ru particles and form RuCu nuclei. At the third stage, Ru atoms grow in one direction on RuCu nuclei, which follows the process reported by Yoon *etc.* that Ru atoms tend to form hexagonal close-packed (hcp) structure^29^ and facilitate the orientation growth. Therefore, with the formation of Ru^0^ and deposition of Cu^0^, the urchin-like RuCu NPs are formed.

**Fig. 5:**
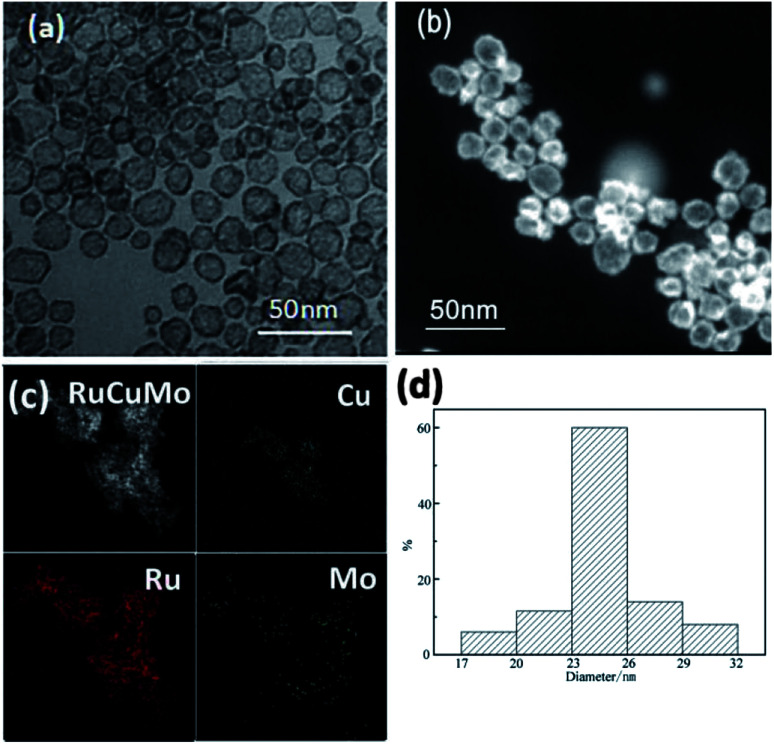
(a) TEM and (b) HAADF-STEM images of hollow RuCuMo nanoparticles; (c) EDX elemental mapping images; (d) size distribution of hollow RuCuMo nanoparticles in (a).

The inexpensive Mo has been reported as co-catalyst to increase NPs' activity.^30^ In order to form a NPs with the tertiary members, Na_2_MoO_4_ was added in the substrate. In the synthesis, we surprisingly found the formation of RuCuMo NPs (Fig. 5). In general, the hollow structure is formed by multiple steps, involving the construction of nanoparticle template, element growing on the surface of the template and the removing of the template. But the complexity and difficulty in the synthesis of this interesting material limited their utility in the practical application.^31,32^ In this work, the hollow nanostructure was obtained by just one-step reduction process and the results were displayed in Fig. 5. Fig. 5c shows the distribution of Ru, Cu and Mo atoms, which confirms the formation of alloyed RuCuMo structure. The HRTEM images of RuCuMo NPs (Fig. S7a, ESI†) displayed the hollow structure and the SAED image also showed the polycrystal structure (Fig. S7b, ESI†). The diameter of the particles can be found in Fig. 5a to be ranged from 17 nm to 32 nm (Fig. 5d). The EDX line scanning analysis of a single particle suggests that Cu and Mo species are mostly concentrated on the shell (Fig. S8, ESI†). The signal difference between the edge and center gives another convincing evidence of the structure with hollow interior.^33,34^ ICP-AES analysis showed that the atomic ratio of Ru : Cu : Mo is 0.75 : 0.20 : 0.05. It can be found that Mo made up a very small proportion in the produced nano particles. This should be caused by the slow reduction rate of Mo species due to the relatively low redox potential of MoO_4_^2−^/Mo (−1.05 V *vs.* SHE), which result in a low content of Mo in the final product.

For the XRD pattern of RuCuMo NPs in Fig. S9 (ESI†), the three peaks were identified as the RuCuMo alloy, but shifted slightly to higher angles compared with the Ru metal standard (JCPDS no 06-0663). This peak shift is possibly resulted from the change in lattice parameters. Replacing the Ru position with Cu and Mo atoms will reduce the unit cell volume,^35,36^ resulting in a lattice parameters reduction. There was no standard JCPDS file can be referred to for the RuCuMo alloy. However, based on the XRD pattern obtained, we believe that the of RuCuMo alloy was formed due to the three characteristic peaks of the synthesized product were all among the three single metal characteristic peaks. The diffraction peaks of RuO_2_ (110) (2*θ* = 28.0°) (JCPDS card no 43-1027) appeared once again, suggesting the presence of Ru oxidation state (RuO_2_).

The NPs can be achieved in 10 min in ternary system, however, it takes 50 min to form NPs in binary system. It suggests that the reduction of the metal precursors can be promoted by the addition of Na_2_MoO_4_. PVP has been reported to be used as the capping agent and weaker reducer in the published works, we found in this work that the addition of PVP in the reaction system could prevent the formed particles from being aggregated (Fig. S10, ESI†), which may be due to the capping and reducing activity as reported.^37,38^

**Fig. 6:**
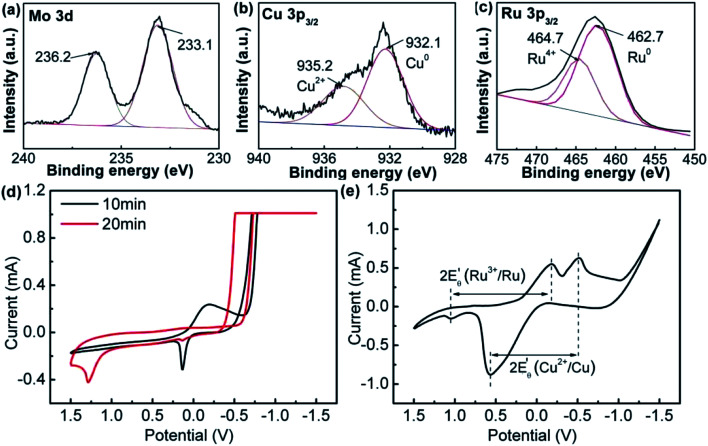
(a) Mo 3d, (b) Cu 2p_3/2_ and (c) Ru 3p_3/2_ XPS spectra of RuCuMo nanoparticles; (d) the CV curves with different RuCuMo nanoparticles sampling time; (e) the electrode potential of Ru^3+^/Ru, Cu^2+^/Cu and MoO_4_^2−^/Mo.

The XPS analysis displayed the bonding state of the metal atoms in RuCuMo NPs. The peaks at ∼233.1 eV and ∼236.2 eV could be assigned to Mo^6+^ state (Fig. 6a), which suggests the oxidation effect (Mo to MoO_3_) on the surface.^39^ Such oxidation of nanosized metals is normal.^40^Fig. 6b shows 36.1% Cu^2+^ in RuCuMo NPs. The Ru^4+^ ratio of RuCuMo NPs was declined to 21.1% from 26.9% of RuCu NPs. It is indicated that, compared with binary RuCu NPs, ternary RuCuMo NPs is more stable. The result indicates the combination of Ru and the modified non-noble transition metals improves the stability due to the electron interaction of the elements.^41^

To understand the formation process of the ternary RuCuMo NPs, CV analysis was performed, as well as the analysis of electrode potential of Ru^3+^/Ru, Cu^2+^/Cu and MoO_4_^2−^/Mo (Fig. 6d and e). The standard redox potential of Ru^3+^/Ru^0^, Cu^2+^/Cu^0^ and MoO_4_^2−^/Mo is 0.68 V, 0.34 V and −1.05 V, respectively,^8,42^ therefore, Ru ion is more easily reduced than the other two metal ions. However, according to Fig. 6d and S6 (ESI†), the CV curve at 10 min is consistent with that of Cu NPs, then the CV curve at the 20 min agrees with RuCu NPs, which suggests the Cu nuclei is obtained at the initial stage, and then Ru^3+^ is reduced. In addition, the analysis of the electrode potential (
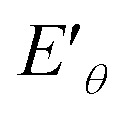
) (Fig. 6e) shows that 
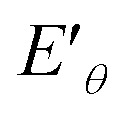
 of Ru^3+^/Ru^0^ don't be changed (
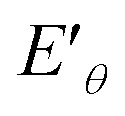
 = 0.68 V) and 
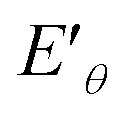
 of Cu^2+^/Cu^0^ is increased to 0.59 V from original 0.34 V, which also suggests that the reduction of Cu^2+^ in this system could be easier. The reduction of MoO_4_^2–^/Mo was not seen in the process (Fig. 6e and S11c, ESI†), which suggests that it is difficult to get reduced in this system due to its redox potential. Therefore, the reduction of the ions is in the order of Cu^2+^, Ru^3+^ and then MoO_4_^2−^.

**Fig. 7:**
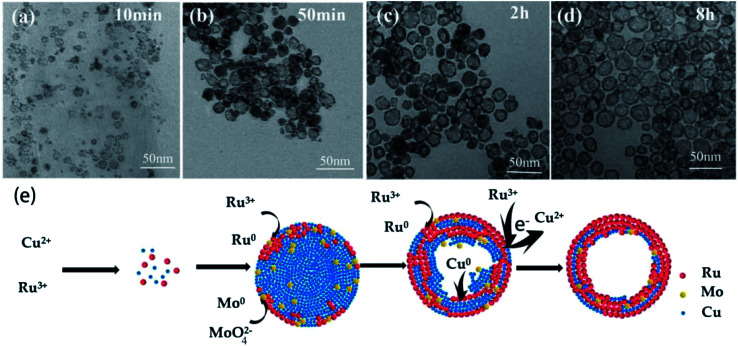
Representative TEM images of the intermediates obtained at different reaction time during the synthesis of hollow RuCuMo nanoparticles: (a) 10 min, (b) 50 min, (c) 2 h, and (d) 8 h, respectively. (e) Schematic illustration of the formation process of RuCuMo trimetallic nanoparticles.

In order to further gain insight into the morphological evolution of RuCuMo NPs, typical TEM analysis of the samples at 10 min, 50 min, 2 h and 8 h respectively was performed (Fig. 7). At the initial stage, the NPs with the size of 3–10 nm are obtained at 10 min (Fig. 7a). The hollow particles were formed at 50 min (Fig. 7b) and the hollow structure with size of ∼23 nm were built at 8 h (Fig. 7d). Then the shape and size of the final products did not change with the reaction time.

Based on the discussion above, the formation mechanism of RuCuMo NPs is proposed in Fig. 7e. At the first stage, Cu and Ru precursors are reduced by C_6_H_5_CH_2_OH. Due to the combination of Ru^3+^ and MoO_4_^2−^ at early stage, Cu^2+^ is reduced mainly in the initial phase. At the second stage, Ru atoms are preferentially positioned on the outer part of the formed Cu@Ru nano-nuclei because Ru atom is bigger in size than Cu atom. The reduction process is always accompanied with the diffusion process,^43^ so the small proportion of Mo atoms are spread in the inner part of the hollow Cu@Ru particle and be intercepted among Cu and Ru atoms. At the third stage, Cu atoms are *in situ* oxidized by Ru^3+^ (Cu (s) + 2Ru^3+^ → 3Cu^2+^ + Ru (s)) due to the galvanic replacement reaction.^44^ Finally, the oxidized Cu atoms are removed from the solid NPs by dissolution into the solvent and the pits are filled with Ru atoms. The removal of Cu atoms makes the particle hollow inside.

## 4. Conclusions

In conclusion, we have successfully synthesized urchin-like RuCu and hollow RuCuMo NPs by one-pot reduction method. The morphology of the NPs is dependent upon the precursors in the reaction system and their reduction sequences. Compared to previously reported multi-step synthetic routes, the developed method here is much simpler. It is believed that cyclic voltammetry analysis can provide a profound information for synthesis of NPs.

## Conflicts of interest

There are no conflicts to declare.

## Supplementary Material

RA-008-C8RA01261J-s001
