# Supplementary material for: Facile synthesis of urchin-like RuCu and hollow RuCuMo nanoparticles and preliminary insight to their formation process by cyclic voltammetry
Source: RSC Adv. 2018 Apr 17;8(26):14138–43. doi: 10.1039/c8ra01261j (PMC9079869; doi:10.1039/c8ra01261j)
Supplement: RA-008-C8RA01261J-s001 [file RA-008-C8RA01261J-s001.pdf]

Electronic Supplementary Material (ESI) for RSC Advances.
This journal is © The Royal Society of Chemistry 2018

Electronic Supplementary Information

Facile synthesis of urchin-like RuCu and hollow RuCuMo nanoparticles and preliminary insight to their formation process by cyclic voltammetry

Yanna Song[a], Jingcheng Sun[a], Yanru Zhang[a], Bingxin Wang[a], Qiang Li[*c] and Yongming Fan [*ab]

[a] MOE Engineering Research Center of Forestry Biomass Materials and Bioenergy, Beijing Forestry University, Beijing, 100083, China

[b] Key Laboratory of Lignocellulosic Chemistry, Beijing Forestry University, Beijing, 100083, China

[c] College of Science, Beijing Forestry University, Beijing 100083, China

[*] Correspondence: liqiang@bjfu.edu.cn (Q. L.); fanym@bjfu.edu.cn (Y. M.);

Tel. +86-137-18679671 (Q. L.); +86-185-15301003 (Y. M.)

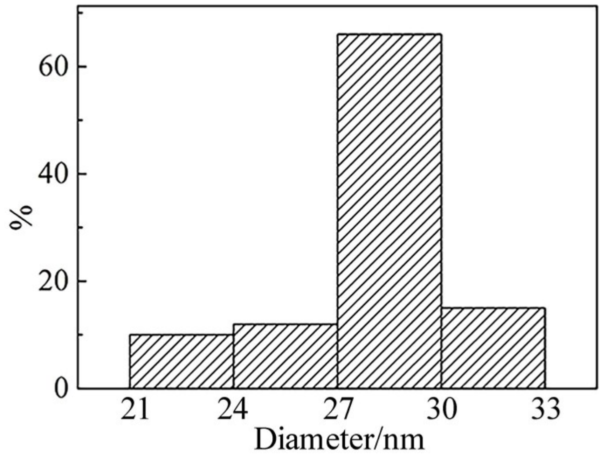

Fig. S1 Size distribution of the urchin-like RuCu nanoparticles in Fig. 1(a).

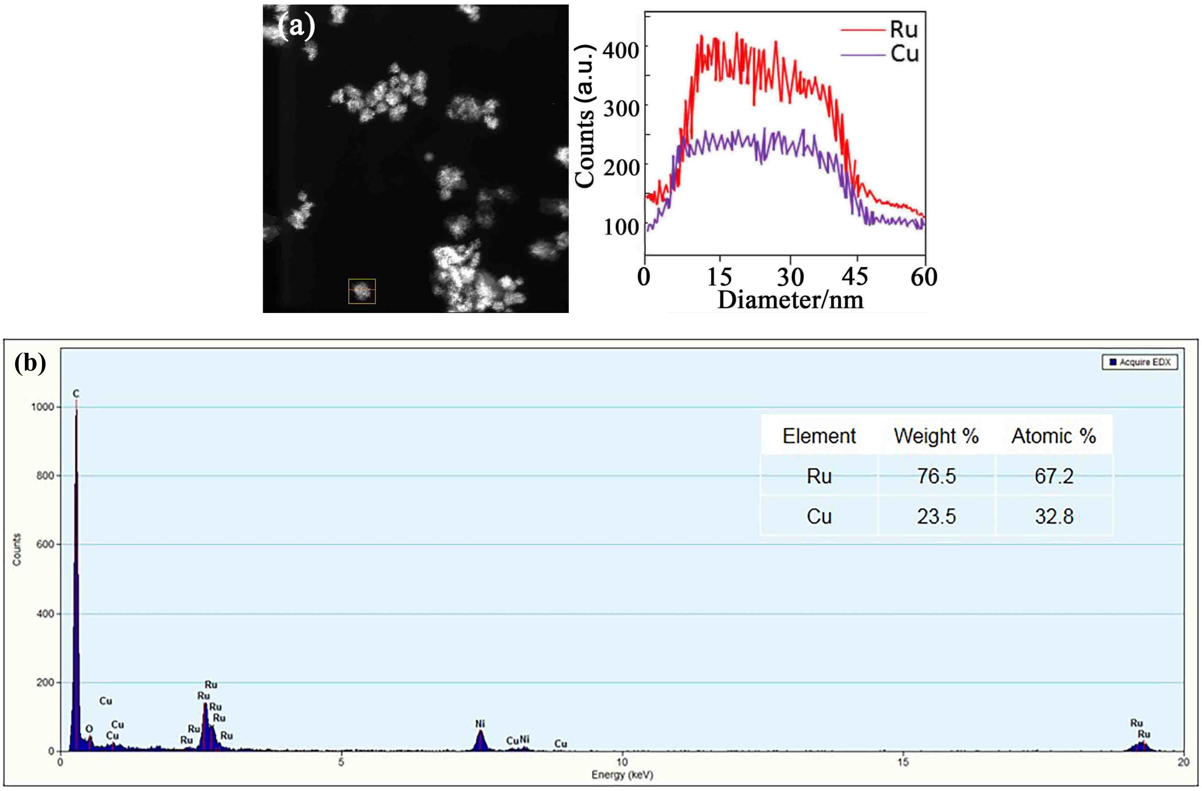

Fig. S2 (a) The cross-sectional compositional line profiles of urchin-like RuCu nanoparticles; (b) EDX patterns of RuCu NPs.

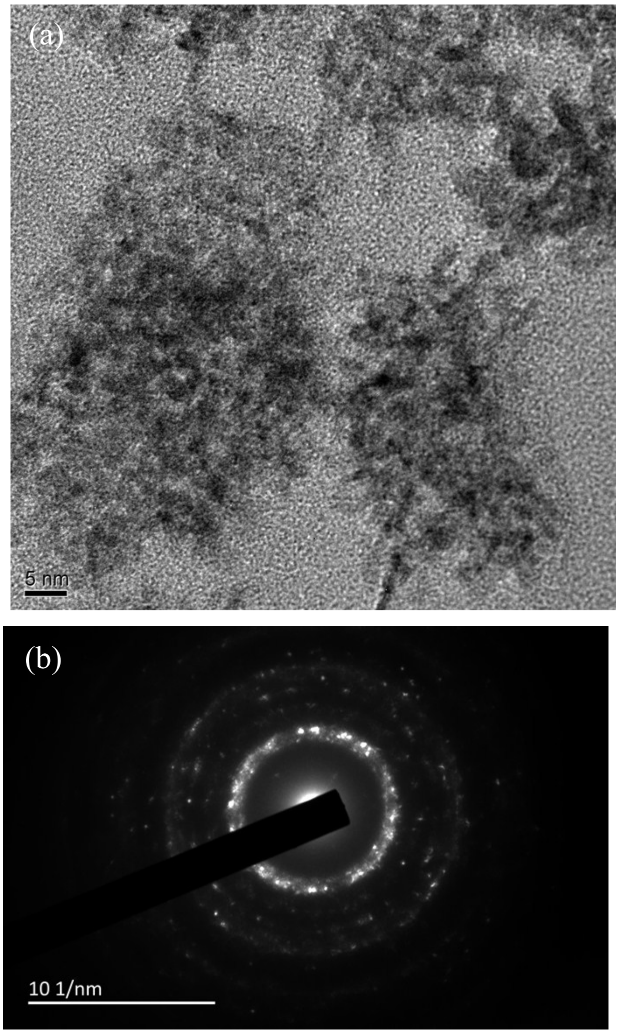

Fig. S3 (a) HRTEM images and (b) SAED image of RuCu NPs.

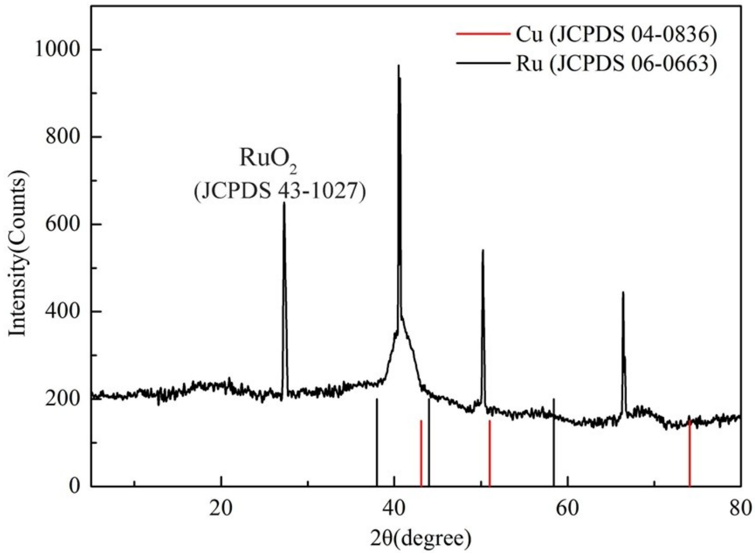

Fig. S4 XRD pattern of RuCu NPs.

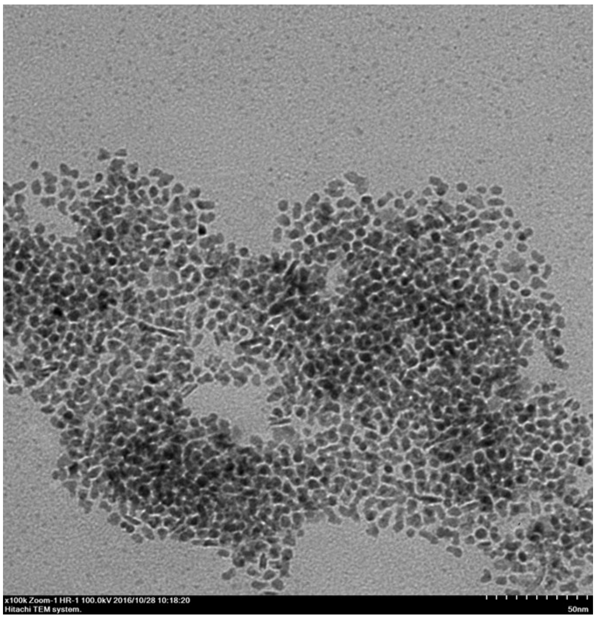

Fig. S5 The TEM images of NPs in only Ru precursor system.

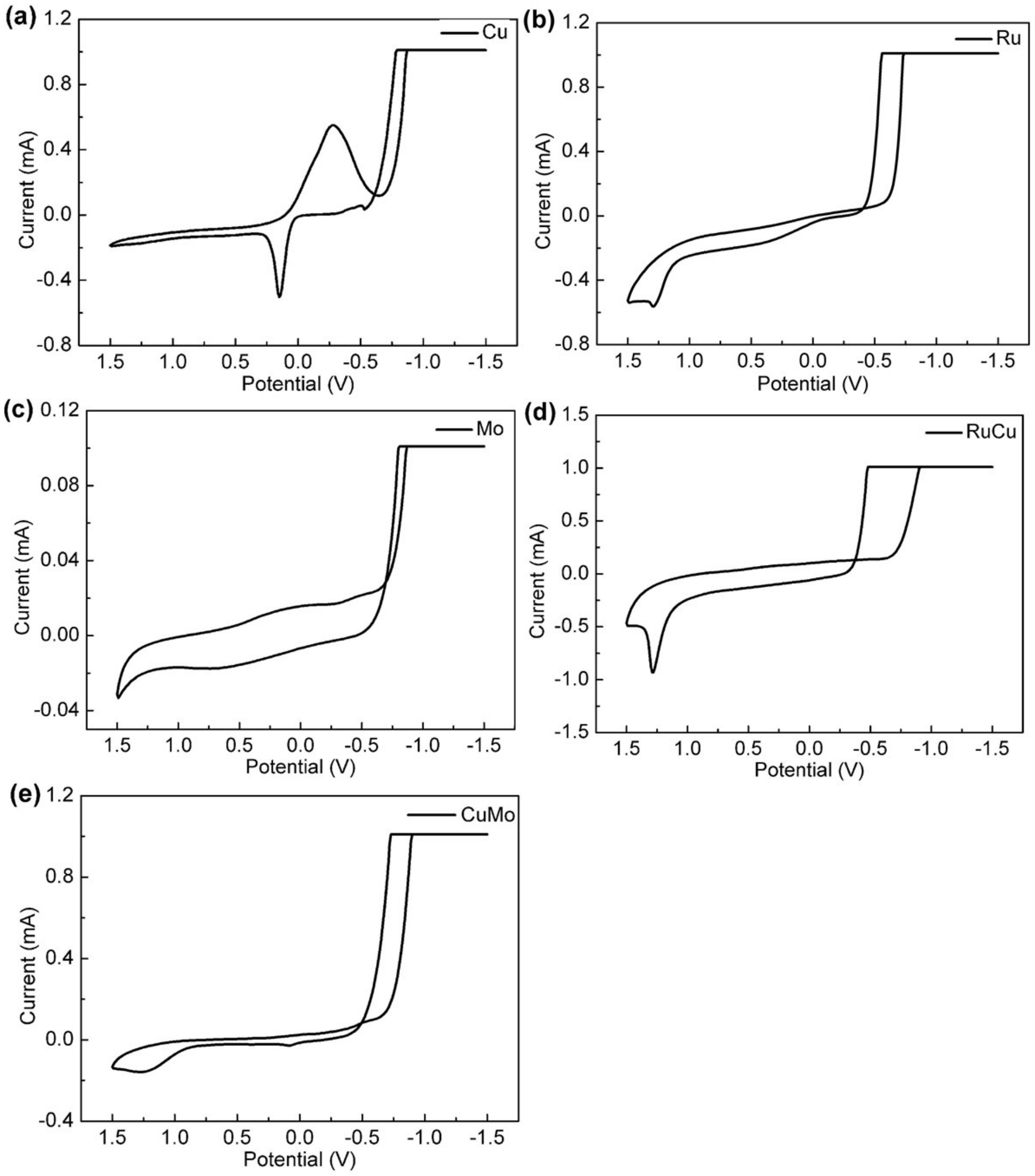

Fig. S6 The CV curves of different nanomaterials (a) Cu single metal nanomaterials; (b) Ru single metal nanomaterials; (c) Mo single metal nanomaterials; (d) RuCu bimetallic nanomaterials; (e) CuMo bimetallic nanomaterials.

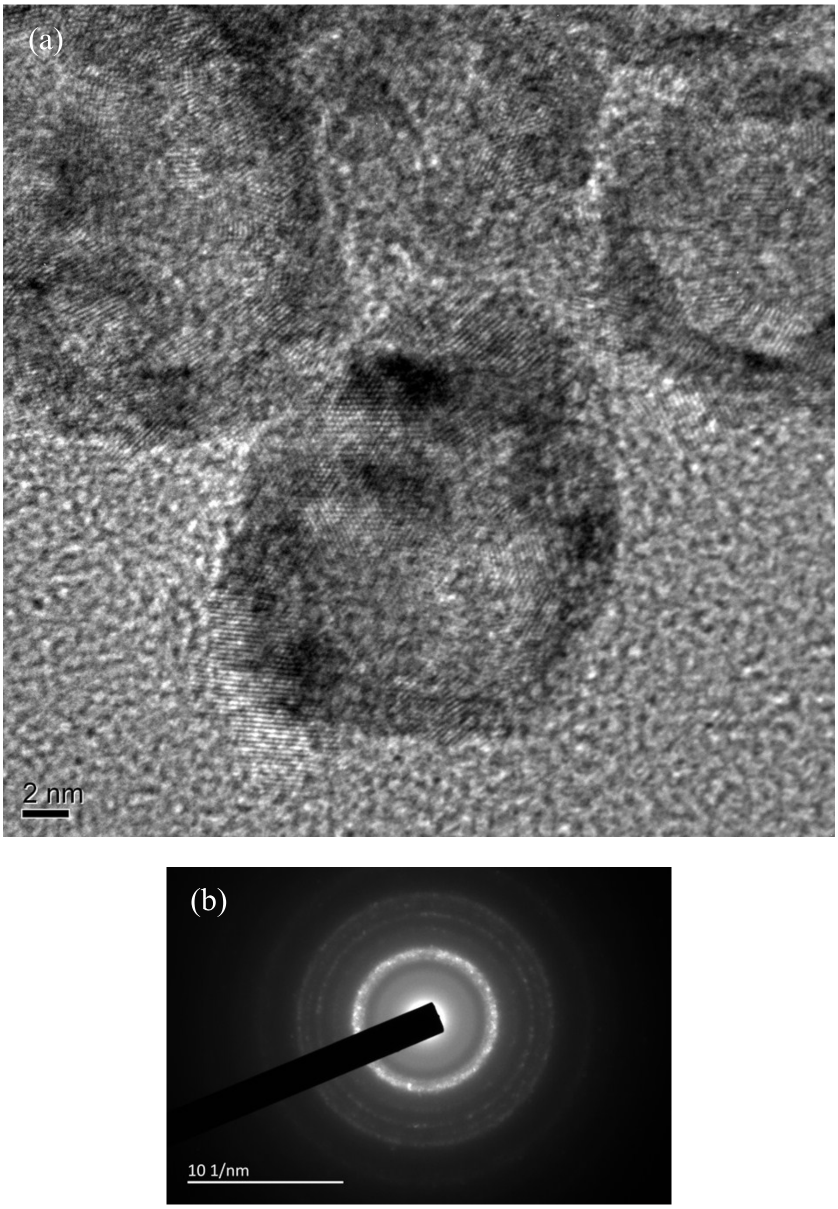

Fig. S7 (a) HRTEM images and (b) SAED image of RuCuMo NPs.

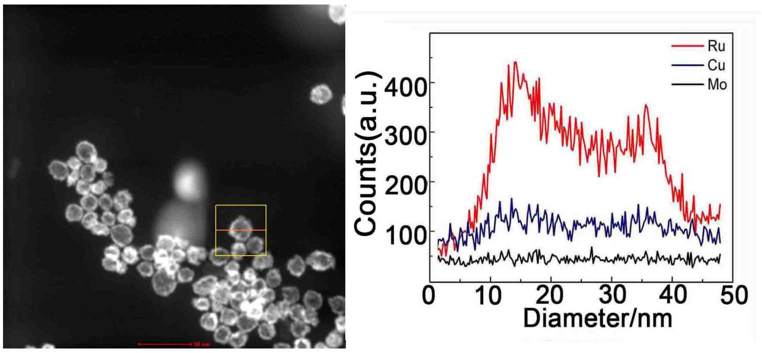

Fig. S8 The cross-sectional compositional line profiles of hollow RuCuMo nanoparticles.

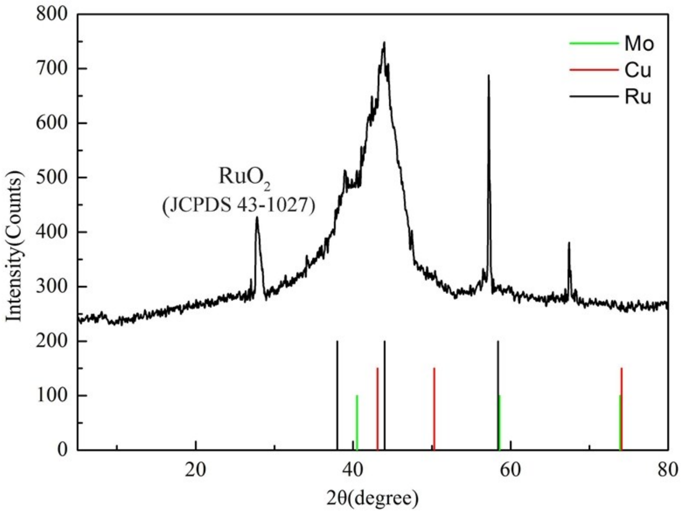

Fig. S9 XRD pattern of RuCuMo NPs.

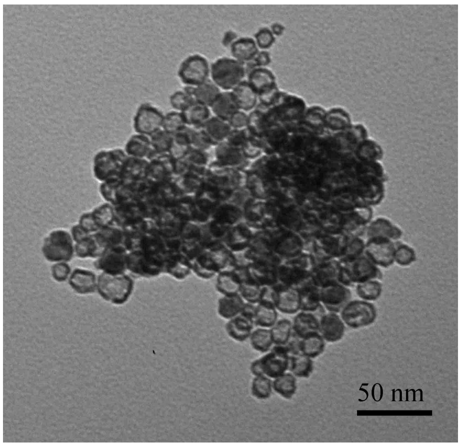

Fig. S10 The TEM image of RuCuMo NPs prepared without PVP in the system.

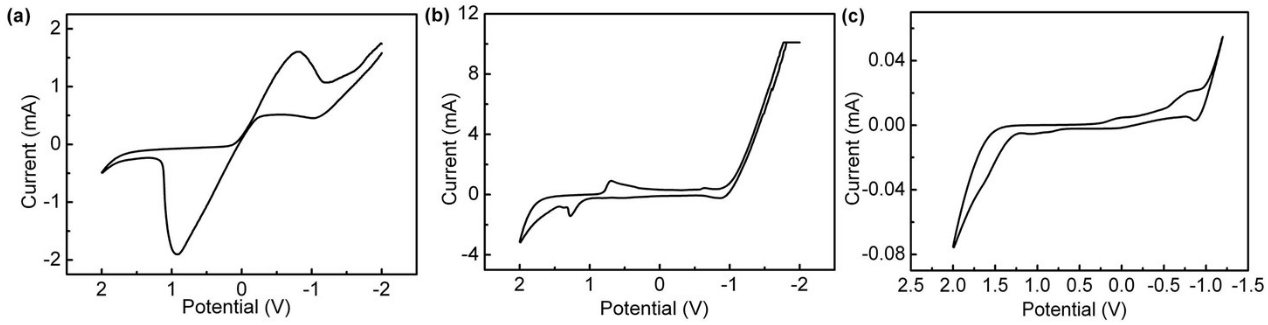

Fig. S11 The electrode potential of single (a) Cu; (b) Ru; (c) Mo in the solution.
